# Hydrophobic interaction governs unspecific adhesion of staphylococci: a single cell force spectroscopy study

**DOI:** 10.3762/bjnano.5.163

**Published:** 2014-09-10

**Authors:** Nicolas Thewes, Peter Loskill, Philipp Jung, Henrik Peisker, Markus Bischoff, Mathias Herrmann, Karin Jacobs

**Affiliations:** 1 Experimental Physics, Campus E2 9, Saarland University, D-66123 Saarbrücken, Germany; 2 Present address: Dept. of Bioengineering and California Institute for Quantitative Biosciences (QB3), University of California at Berkeley, Berkeley, California 94720, USA; 3 Institute of Medical Microbiology and Hygiene, Saarland University, D-66421 Homburg/Saar, Germany

**Keywords:** atomic force microscopy (AFM), force spectroscopy, hydrophobic interaction, single cell, *Staphylococcus carnosus*

## Abstract

Unspecific adhesion of bacteria is usually the first step in the formation of biofilms on abiotic surfaces, yet it is unclear up to now which forces are governing this process. Alongside long-ranged van der Waals and electrostatic forces, short-ranged hydrophobic interaction plays an important role. To characterize the forces involved during approach and retraction of an individual bacterium to and from a surface, single cell force spectroscopy is applied: A single cell of the apathogenic species *Staphylococcus carnosus* isolate TM300 is used as bacterial probe. With the exact same bacterium, hydrophobic and hydrophilic surfaces can be probed and compared. We find that as far as 50 nm from the surface, attractive forces can already be recorded, an indication of the involvement of long-ranged forces. Yet, comparing the surfaces of different surface energy, our results corroborate the model that large, bacterial cell wall proteins are responsible for adhesion, and that their interplay with the short-ranged hydrophobic interaction of the involved surfaces is mainly responsible for adhesion. The ostensibly long range of the attraction is a result of the large size of the cell wall proteins, searching for contact via hydrophobic interaction. The model also explains the strong (weak) adhesion of *S. carnosus* to hydrophobic (hydrophilic) surfaces.

## Introduction

Members of the genus *Staphylococcus* are known to form extremely resistant biofilms, some of which can cause severe infectious diseases [1]. *Staphylococcus carnosus* is an apathogenic member of that genus and has been described first in the early 1980s [2]. The name *Staphylococcus carnosus* reflects its important role in meat production as it reduces nitrate to nitrite and prevents food rancidity by producing the anti-oxidant enzymes catalase and superoxide dismutase [3].

Only recently, the genome of *S. carnosus* strain TM300 has been decoded [4–5]. In contrast to pathogenic staphylococcal species, such as *S. aureus* and *S. epidermidis*, the genome of *S. carnosus* lacks significant amounts of mobile genetic elements, and is poor in repetitive DNA sequences that are thought to facilitate the plasticity of genomes by allowing for enhanced genomic diversification due to recombinational events [5]. Although the *S. carnosus* genome encodes some homologues of adhesion factors found in *S. aureus*, it lacks the majority of adhesive molecules of its pathogenic relative that are thought to be important for the ability of the pathogen to colonize and invade its mammal hosts (reviewed in [1]). Due to its apathogenic properties, and its ability to be transformed with and to express virulence factors of pathogenic staphylococcal species ([6–7]), the strain TM300 is an ideal tool to study the properties of a single virulence factor and its impact on infectivity in this otherwise apathogenic species. Besides this, it has been shown that the survival of bacteria in a food industry environment is strongly related to their efficiency to adhere on abiotic surfaces [8–10]. Therefore, and because adhesion is the first step of the formation of biofilms, the characterization of bacterial adhesion forces has gained increasing importance in recent years [11].

In general, the adhesion of bacteria to a surface is determined by the nature of the bacterium, the surrounding medium, the surface chemistry, and the material composition reflecting the influence of the main interacting forces [12–13]: van der Waals forces, hydrophobic interaction and electrostatic forces. In addition, specific interactions amplify bacterial adhesion whenever corresponding binding partners are available. The adhesion process of microorganisms, such as *S. carnosus*, is usually characterized by using flow chambers [14]. Although flow chamber studies reproduce the natural adsorption process of microorganisms out of fluid flow, it is hard to determine quantitative adhesion force values. The outcome usually results from multiple parallel processes, such as adsorption, desorption, and adhesion. Moreover, results obtained from flow chamber experiments depend on the exact flow conditions of the used chamber [15]. In the last decade, a more quantitative method for measuring bacterial adhesion forces has been introduced: single-cell force spectroscopy is a special mode of an atomic force microscope (AFM) [16] and is optimized to investigate adhesion forces [17–18] of single bacterial cells in a very controlled manner: By using AFM-cantilevers functionalized with single bacteria, “bacterial probes”, force/distance measurements are conducted. To date, single cell force spectroscopy is mostly used for exploring specific adhesion [19]. It is the aim of this study to characterize the unspecific adhesion mechanisms of *Staphylococci*, by using *S. carnosus* as an example, and to clarify the range of the attractive interaction of the cells to surfaces. We use abiotic surfaces in order to rule out effects due to specific interactions and to concentrate on the unspecific interactions of *S. carnosus* to surfaces of variable surface energy. As a unique feature of our study, we are able to probe different surfaces with the exact same bacterial cell. Thereby, we are able to demonstrate the importance of the hydrophobic interaction on the bacterial adhesion process. Moreover, we can measure the adhesion forces that are mediated by bacterial cell wall proteins (and further cell wall components) and test their dependency on the ‘adhesion history’ the cell has experienced before.

## Experimental

### Preparation of the substrates

The hydrophilic substrates used in this study are silicon wafers with a native silicon oxide layer (*d* = 1.7(2) nm) (the number in parentheses denotes the error of the last digit) purchased from Siltronic AG (Burghausen, Germany). In order to remove dirt, the silicon wafers were first immersed for 30 min in fresh 1:1 H_2_SO_4_ (conc.)/H_2_O_2_ (30%) solution, then in boiling deionized water for 90 min, during which the water was changed at least four times. Following a standard protocol, these hydrophilic surfaces can be rendered hydrophobic by the self-assembly of a monolayer of silane molecules (octadecyltrichlorosilane, OTS, Sigma-Aldrich), featuring a CH_3_-tailgroup [20–21]. As has been shown in [21] by perfoming AFM, X-ray reflectometry, ellipsometry, and contact angle measurements, this protocol enables the preparation of a self-assembled monolayer (SAM) with a thickness of about 2.6 nm and an *rms* roughness below 0.2 nm. In [21] it was shown that the SAM is hydrophobic, homogeneous, dense, upright and in all-trans configuration. The contact angles, surface roughnesses and surface energies for hydrophilic and hydrophobic wafers are given in Table 1 and streaming potential measurements reveal that both surfaces are negatively charged at the used pH of 7.3 (Table 1). For this study, OTS surfaces of the same batch as in [21] have been used. Prior to the AFM force spectroscopy experiments with bacterial cells, both types of surfaces were immersed in PBS buffer.

**Table 1:** Parameters of the model substrates: Root mean square (*rms*) roughness, advancing (adv) and receding (rec) contact angles Θ of water, surface energy γ (values taken from [21]) and surface charge as revealed by streaming potential measurements at pH 7.3 [22].

surface	*rms* (nm)	Θ_adv_	Θ_rec_	γ (mJ/m^2^)	streaming potential (mV)

hydrophilic	0.09(2)	7(2)°	compl. wetting	64(1)	−104.4(1)
hydrophobic	0.12(2)	111(1)°	107(2)°	24(1)	−80.0(1)

### Bacteria

For the experiments, freshly prepared exponential phase *S. carnosus* strain TM300 cells were used [4]. The bacteria were cultured on blood agar plates and transferred into 5 mL of TSB medium for 24 h at 37 °C. Before the experiments, 100 μL were transferred into 4 mL fresh TSB medium and cultured for 2.5 h at 37 °C and 150 rpm. To remove extracellular material, the bacteria were washed four times with phosphate-buffered saline (PBS, pH 7.3, ionic strength 0.1728 mol/L at 20 °C), each with 1 mL. Then, the bacteria were either used immediately or stored less than two hours at 4 °C. For the preparation of the bacterial AFM probes, bacterial solution was again diluted 1:6.

### Preparation of the bacterial probes

Bacterial probes are based on tipless cantilevers (MLCT-O, Bruker, Billerica, MA, USA) with a nominal spring constant of 0.03 N/m. After the cantilevers were cleaned in an air plasma, they were vertically immersed into a solution of 4 mg/mL dopamine hydrochloride (99%, Sigma-Aldrich) in 10 mM TRIS-buffer (pH 7.9 at 22 °C) and kept at 4 °C in the fridge for 50 min. The cantilevers were then carefully rinsed in deionized water to remove unbound dopamine and dried under a laminar flow bench for at least one hour. The poly(dopamine)-covered cantilever was then inserted into the Bioscope Catalyst cantilever holder for measurements in liquids (Bruker, Billerica, MA, USA), mounted onto the AFM. Subsequently, it was calibrated in liquid by using the thermal tune technique [23], which allows for the calculation of the individual spring constant of the cantilever.

Afterwards, holder and cantilever were placed into a micromanipulation system (Narishige Group, Japan). The cantilever thereby is in the horizontal position with the functionalized side facing down. By using the micromanipulator, holder and cantilever were lowered and the cantilever dipped into a droplet of diluted bacterial solution (see above), which was previously placed on a polystyrene petri dish. Under the inspection of an inverted optical microscope, the cantilever was placed on top of an isolated bacterium, briefly and carefully tapped onto the cell, and immediately pulled back again. In order to keep the applied force low, the deflection of the cantilever was monitored by the light reflection off the cantilever and kept constant during motion. Care was taken to position the bacterium as close as possible to the end of the cantilever (not further away than two bacterial diameters) to safely exclude cantilever/substrate interactions.

**Figure 1:**
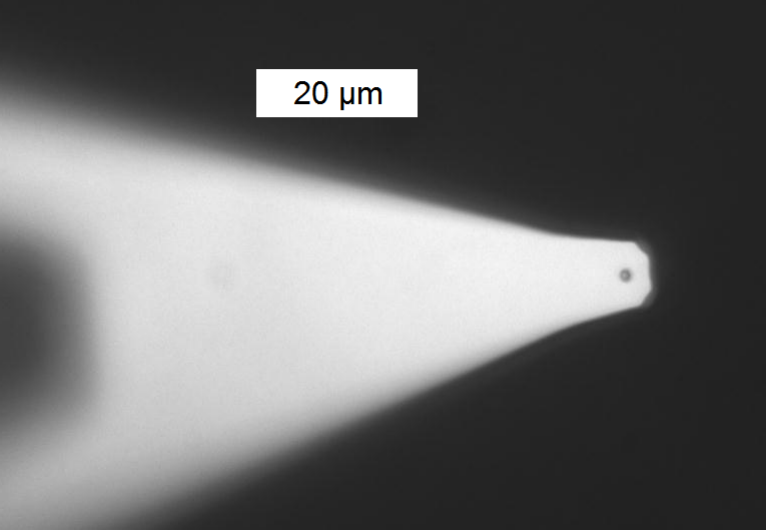
Optical image of a single *S. carnosus* bacterium immobilized by (poly)dopamine on a tipless cantilever.

The successful fixation of a single bacterium at the front end of the cantilever was confirmed with the inverted microscope, c.f. Figure 1. Subsequently, cantilever (and holder) were withdrawn from the bacterial solution, whereby a droplet clings to the cantilever, preventing the bacterium from drying. Cantilever and holder were then carefully transferred to the AFM and immediately immersed into the PBS filled petri dish containing the hydrophilic and hydrophobic substrates.

### Single cell force spectroscopy measurements

Single cell force spectroscopy measurements were performed by using a Bioscope Catalyst (Bruker, Billerica, MA, USA). The deflection of the cantilever is recorded during the approach and retraction of the bacterial probe to and from the surface. The deflection data was converted into force values by means of the spring constant of the cantilever, determined as described above. The approach is performed until a certain repulsive force is reached (“force trigger”), typically 150 pN in this study, if not indicated otherwise. Experiments were performed in 6 mL PBS at 20 °C and with tip velocities between 400 nm/s and 2400 nm/s over a total distance of typically 800 nm. (It is important to note here that the tip velocity describes the velocity of the piezo drive that controls the *z*-movement of the cantilever, which, however, is not necessarily identical with the velocity of the bacterium, especially at retraction.)

For each parameter set, at least 30 force/distance curves were recorded, each on a different surface spot to avoid systematic errors due to local irregularities of the surface or contamination due to preceding adhesion events. If two surfaces were to be compared, the first set of 30 force/distance curves was taken on one surface followed by a series of 30 curves on the second surface, then again 30 on the first surface. By doing this, we took control of the reproducibility of the measurements and can rule out systematic errors like the degradation of the bacterial probe.

**Figure 2:**
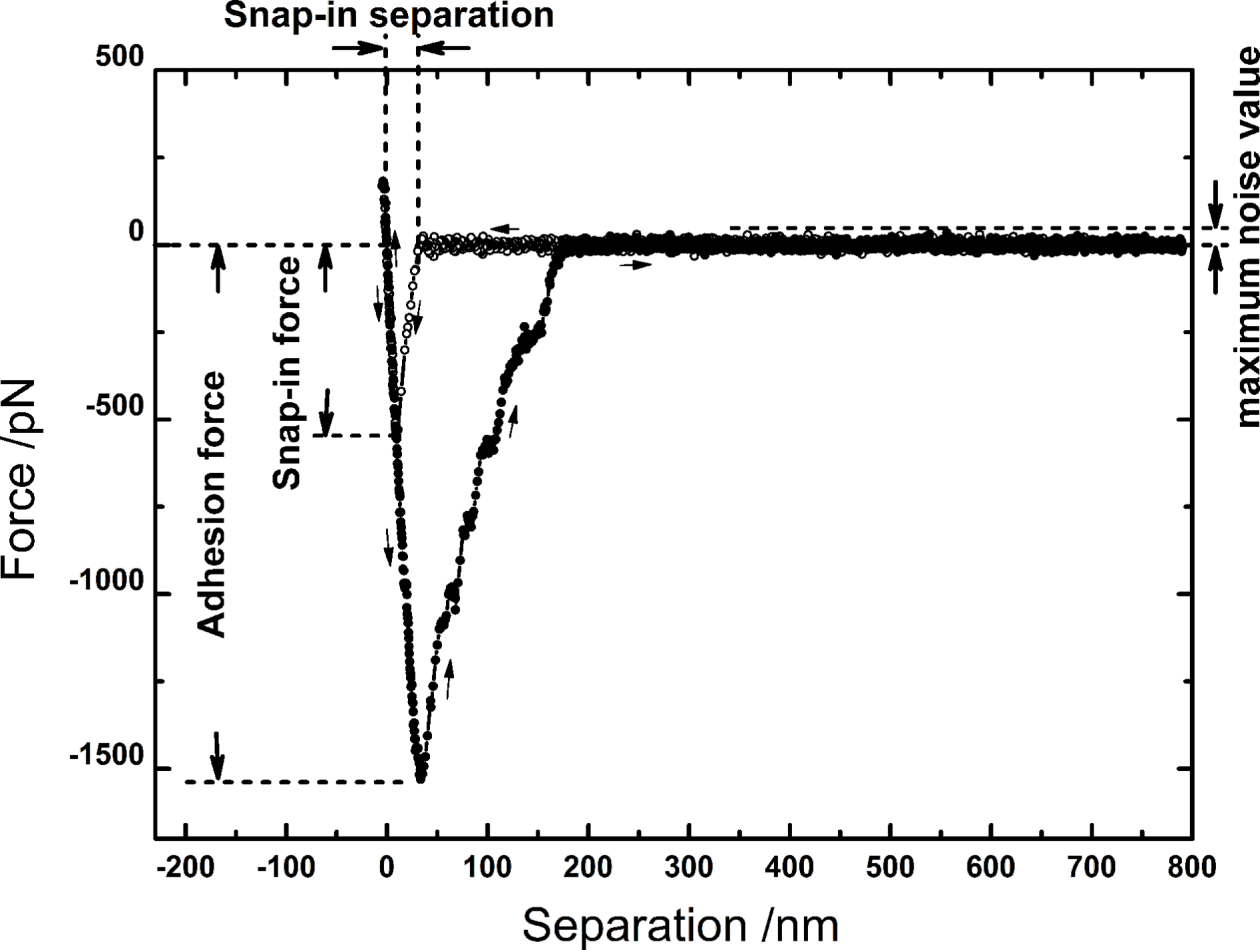
Typical force/distance curve, taken with a single *S. carnosus* probe on an OTS-covered (hydrophobic) Si wafer. By analyzing the curve, the adhesion force, the “snap-in force” and the “snap-in separation” can be obtained, the description of the latter is given in the text. The arrows indicate the direction of motion of the piezo drive.

A typical force curve is shown in Figure 2. Upon approach, a jump-to-contact (“snap-in”) event can be observed, followed by a steep rise of the force, indicating bacterium/surface contact. Since the exact contact formation and mechanics between the bacterial surface and the solid substrate is unclear, it is hard to make predictions on the shape of the force/distance curve. Upon retraction, the force/distance curve exhibits first the same steep slope as upon approach, yet, due to adhesive forces, a deep global minimum is recorded. Further retraction provokes a loss of contact (“jump-off contact”). In the repulsive regime (F > 0), a force of 150 pN is not enough to deform the bacterium: With a force trigger of 150 nN, only an indentation of ca. 10 nm is reached (cf. Figure 2). Therefore, very likely, only components of the cell wall are elastically deformed.

Three general measures allow for a comparison of the adhesion process: Inspecting the approach curve, the “snap-in” event is characterized by the depth of the global minimum, called “snap-in force” and by the “snap-in separation”, defined as the separation at which the deflection reaches 150% of the maximum baseline noise value (typically between 30 and 50 pN), c.f. Figure 2. The snap-in separation serves as a hands-on measure for the determination of the width of the snap-in event. Distances are measured relative to the point of zero force [24]. From the retraction curve, the adhesion force is taken as the depth of the global minimum [24]. Since in some cases, the overall adhesion force decreased after more than about 150 force/distance curves (possibly due to stress applied by the large number of adhesion events), the number of force/distance curves per bacterium was always kept below 150. The bacterium was never “lost” during the experiments, it was safely secured to the cantilever.

**Figure 3:**
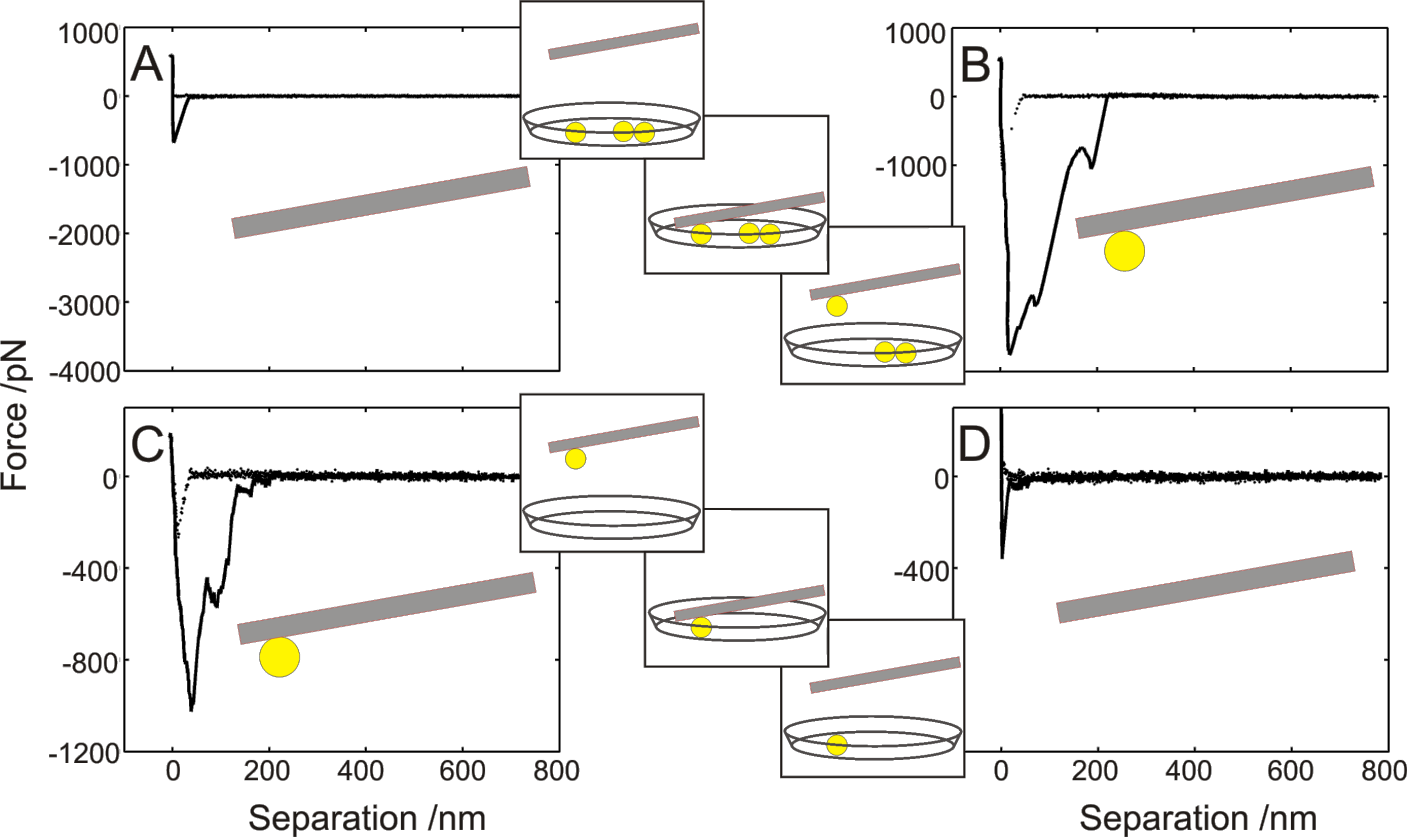
Test of artifacts of AFM force/distance curve with and without bacterium, each taken under the same conditions: (A) Bare (poly)dopamin-coated cantilever force/distance curve. (B) The same cantilever as in (A), yet after attachment of a bacterium. (C) A second cantilever with bacterium and its characteristic force/distance curve. (D) Force/distance curve after detaching the bacterium of the experiment shown in (C).

As a control for the specificity of force/distance curves for bacterial adhesion and to demonstrate that the cantilever does not influence bacterial adhesion, several experiments have been performed with a bare, (poly)dopamin-coated cantilever: Figure 3 displays the difference between the cantilever adhesion signal of the bare (poly)dopamin-coated cantilever (Figure 3A), and the very same cantilever with the bacterial cell attached (Figure 3B). Reversely, after a force/distance curve with an attached bacterium (Figure 3C), the bacterium was removed (by pressing it very hard to a solid substrate followed by shearing it off with a micromanipulator) and another force/distance curve of only the cantilever was recorded (Figure 3D). Clearly, the force/distance curves without bacterium exhibit (nearly) no snap-in event, and during retraction, only a small adhesion peak occurs and the further retraction curve is smooth without the characteristic jumps in the case of an attached bacterium.

## Results and Discussion

**Figure 4:**
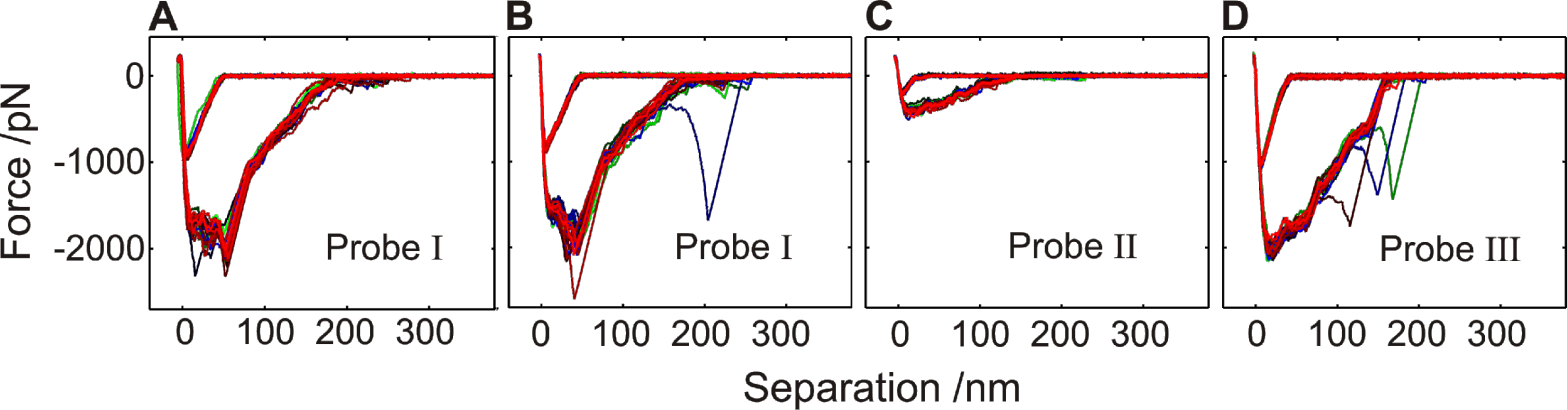
A–D: Overlay of 30 force/distance curves of three individual *S. carnosus* probes I, II and III on hydrophobic Si wafers, each with a force trigger of 150 pN. Between the experimental series shown in A and B, a set of 30 force/distance curves was taken with the identical bacterial probe on a hydrophilic Si wafer (not shown), yet the characteristic form of the second set of force/distance curves did not change significantly.

First, we will concentrate on the robustness of single cell force microscopy. Each individual *S. carnosus* probe achieves a characteristic force/distance curve that can be reproduced numerous times (Figure 4). Differences from curve to curve occur occasionally (about 4 out of 30), yet span mostly only over one section of the retraction curve, otherwise reproducing the rest of the curve. This holds true even if a set of 60 force/distance curves on a hydrophobic wafer is interrupted by the recording of a set of 30 curves on a hydrophilic wafer: The first 30 curves on the hydrophobic wafer are shown in (Figure 4A), the second set of 30 curves is displayed in (Figure 4B), the curves on the hydrophilic wafer are not shown, since they do not differ from those shown in (Figure 7B). The characteristic features of the first set of curves on the hydrophobic wafer is perfectly reproduced by the second set, though in between, the surface energy of the adhesion partner (the hydrophilic surface) was reduced by a factor of three (c.f. Table 1). Hence, as the shape of the force/distance curves is that robust (surviving 150 contact events and surviving even a change of the type of substrate), these results already demonstrate that force/distance measurements are characteristic for the individual bacterial probe and are therefore most suitable to characterize bacterial adhesion.

**Figure 5:**
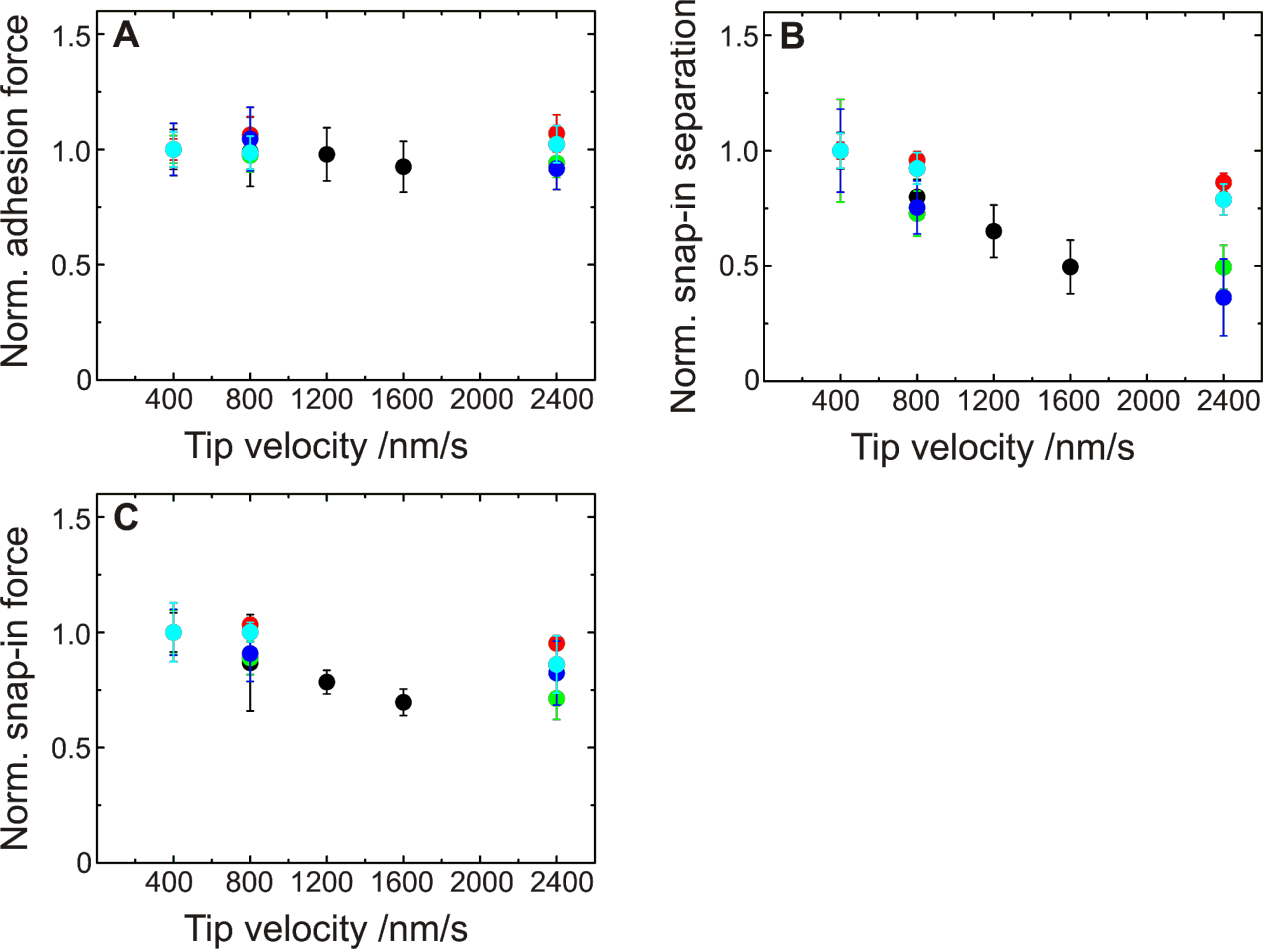
Influence of the tip velocity on the adhesion of *S. carnosus* shown for five different cells. For each bacterium, values for adhesion force (A), snap-in separation (B) and snap-in force (C) are shown. The values are normalized to the value of the measurement with the lowest tip velocity (400 nm/s). Different colors represent different bacterial probes.

Next, we explore the influence of the AFM force spectroscopy parameters on the force/distance curves. The tip (or rather the piezo drive) velocity was varied between 400 nm/s and 2400 nm/s, yet no significant influence on the adhesion force was recorded (Figure 5A). By varying the tip velocity, we implicitly varied the time the bacterium is enabled to gain contact to the surface. Within the range probed, the contact time (estimated to be of the order of a fraction of a second) does not influence the adhesion force. The snap-in separation, however, decreases with increasing tip velocity (Figure 5B), as does the snap-in force (Figure 5C), which is a first indication to a time-dependent contact-process, which will be detailed in the following. For further measurements, a tip velocity of 800 nm/s is used, since for that speed, one force/distance curve of 800 nm ramp size takes 0.5 Hz, which is a convenient frequency and corresponds to frequencies used in other studies [25].

**Figure 6:**
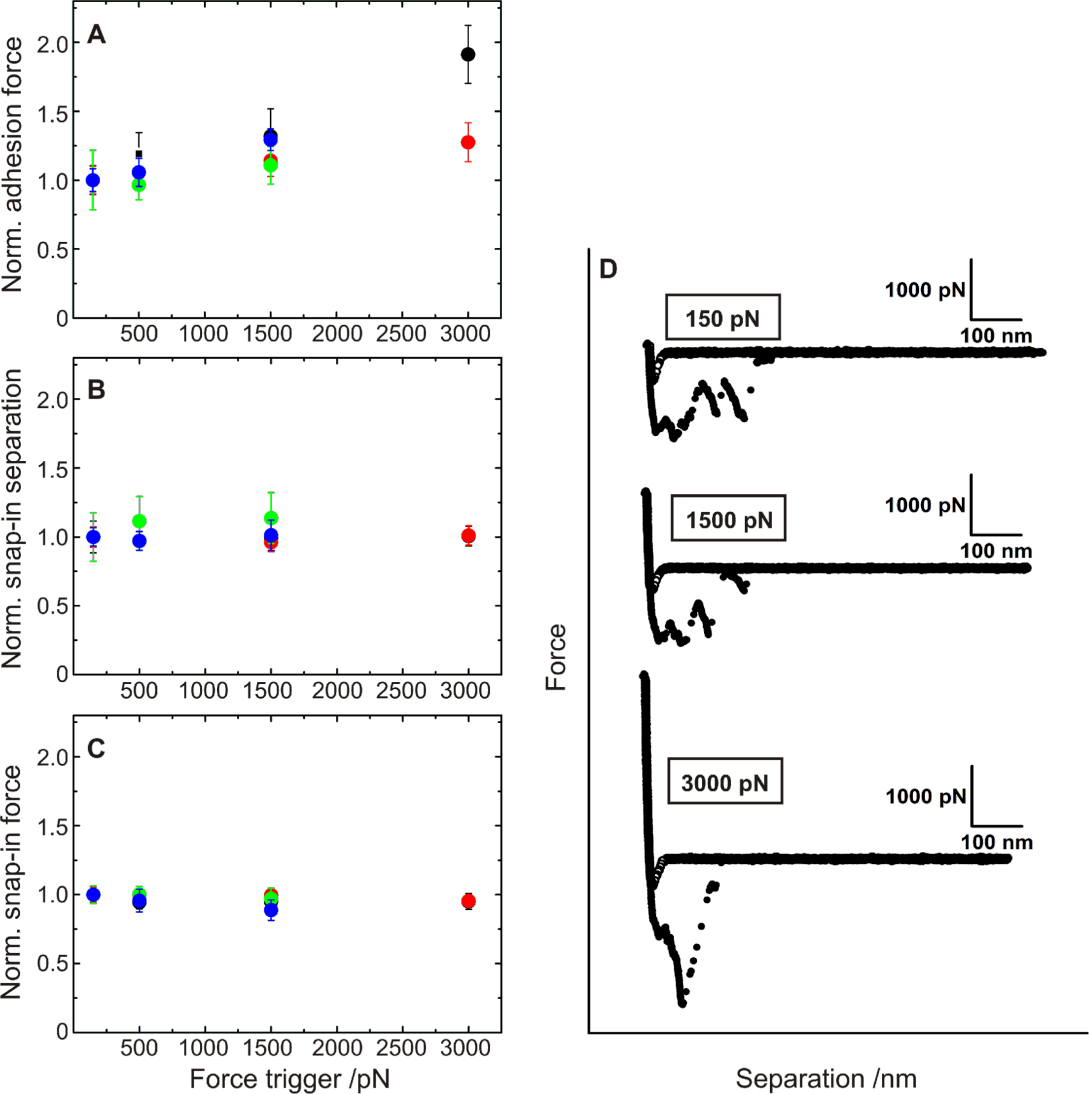
Influence of the force trigger on the adhesion of *S. carnosus*. A–C: For four bacterial probes, values for adhesion force, snap-in force, and snap-in separation are normalized to the value of the measurement with the lowest force trigger (150 pN). D: Exemplary force/distance curves for the exact same bacterial probe for three different force triggers. Different colors represent different bacterial probes.

Increasing the force trigger results in a slight increase of the adhesion force (Figure 6A), whereas the snap-in separation as well as the snap-in force remained constant (Figure 6B and Figure 6C). In the following, the lowest force trigger of 150 pN was used in order to mimic the natural adhesion process of the bacterium in planktonic state.

**Figure 7:**
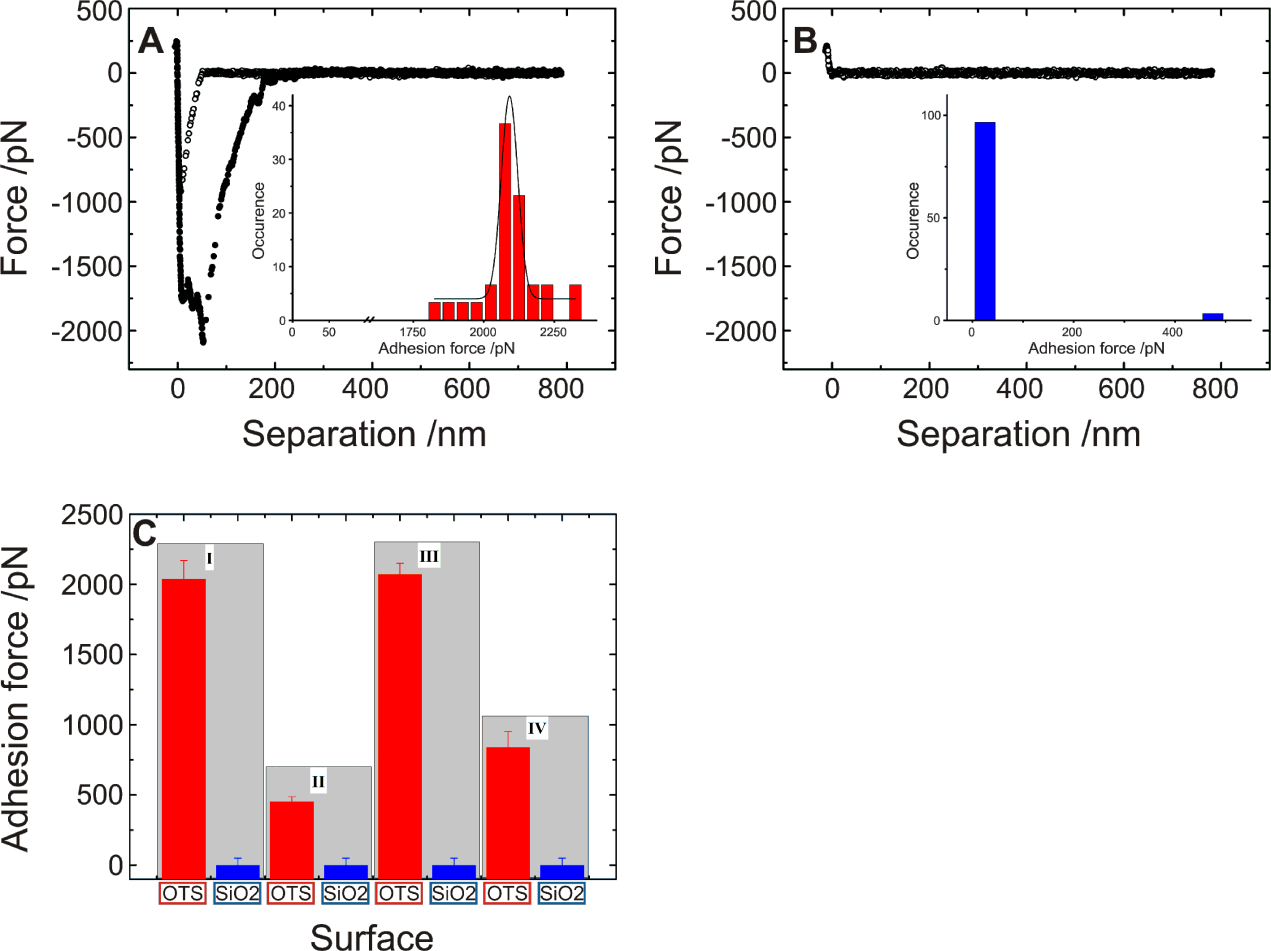
Exemplary force/distance curve taken with a single *S. carnosus* probe on (A) a hydrophobic OTS-covered Si wafer and on (B) a hydrophilic Si wafer. The insets summarize the results of the adhesion force of 30 force/distance curves. (C): Mean adhesion force and standard deviation showing the result of at least 60 force/distance curves of four *S. carnosus* (I–IV) measured on hydrophobic OTS-covered and on hydrophilic Si wafers, covered by a natural SiO_2_. Each of the four bars represents measurements of the exact same bacterial probe.

With the parameters for single cell force spectroscopy as detailed above, we can now specify the large differences in the adhesion of *S. carnosus* to hydrophobic and to hydrophilic surfaces, c.f. Figure 7A and Figure 7B: On the hydrophobic surface, a clear snap-in event is detectable, followed by a large adhesive peak upon retraction. On the hydrophilic surface, however, neither of the two can be recorded. It is important to note that the two curves depicted in Figure 7A and Figure 7B were taken under identical external conditions (temperature, buffer), the same AFM force spectroscopy parameters (force trigger, tip velocity) and, most significantly, were recorded with the same bacterium. Figure 7C demonstrates the individual adhesive properties of different bacteria. Gray bars comprise data of the exact same bacterial probe on the two different surfaces. The data of Figure 7A and Figure 7B are taken from the set of curves summarized in bar I.

Obviously, the adhesive mechanism of *S. carnosus* on hydrophobic OTS surface differs strongly from that on a hydrophilic Si wafer, as the adhesion of *S. carnosus* to hydrophilic Si wafers is barely resolvable. From force spectroscopy measurements with multiple bacteria as AFM probes (30–50 bacteria), we learned that on hydrophilic silicon oxide surfaces, the adhesion force is roughly an order of magnitude lower than on OTS-Si wafers [13]. Hence, the adhesion force of a single bacterium on a hydrophilic surface is expected to be below the experimental resolution (about 50 pN), which explains the present results.

What is the difference of bacterial adhesion to hydrophilic or hydrophobic surfaces? Adhesion is the sum of all forces between the interacting partners. In our case, van der Waals and electrostatic forces as well as forces due to the hydrophobic interaction are involved [26]. Since hydrophilic and hydrophobic Si wafers differ in composition only by a 2.6 nm thin OTS-monolayer on the surface, the van der Waals forces are nearly identical [13,27]. Forces due to electrostatic interactions between the negatively charged bacterium and the two types of wafer surfaces, which are both negatively charged (Table 1), are repulsive. Since the streaming potential is 20% more negative on the hydrophilic Si wafer, different electrostatic interactions give rise to a difference of adhesion forces of only a factor of 1.2, yet we record differences in the range of factors 10 to 40 (Figure 7C). Therefore, we hypothesize that the adhesion of *S. carnosus* is governed by hydrophobic interaction.

Inspecting the snap-off event in more detail, not only the large extent is striking but also the reproducible, stepwise reduction of the recorded force (see Figure 4). This is a strong indication of a reversible fold-and-stretch mechanism of the involved macromolecules. It is known that the bacterial surface is covered by a variety of proteinaceous and non-proteinaceous polymers that can mediate adhesion (adhesins) [28–29]. The form of the retraction part can therefore be explained by a parallel and simultaneous stretching of cell wall proteins tethered to the surface as the piezo retracts [25,30].

Proteins are known to adsorb differently to hydrophilic and hydrophobic surfaces since the hydrophobic interaction can induce intramolecular conformational transitions and change the orientation of hydrophobic side groups of proteins [31–35]. This has also been shown by surface forces apparatus (SFA) experiments [36–38]. The range of the hydrophobic interaction depends on the correlation length of water molecules, which is below 1 nm [39]. Therefore, bacterial surface proteins have to come that close to the OTS surface in order to interact attractively. The SFA studies showed that the more hydrophobic the interacting partners (protein/surface) are, the stronger is the adhesion force.

An influence of further nonproteinaceous cell wall components (like teichoic acids) cannot be excluded and it will depend on the hydrophobicity of these components. However, proteins will play the key role in adhesion to hydrophobic surfaces due to their strong hydrophobic parts. Therefore, we will only talk about cell wall proteins in the following, but are aware of the fact that also other hydrophobic macromolecules may contribute to adhesion. For *S. aureus* for example, teichoic acids are reported to be strongly hydrophilic [40]. Our model of bacterial adhesion, which will be proposed in the following, however, is not depending on the exact type of adhesive mediator.

**Figure 8:**
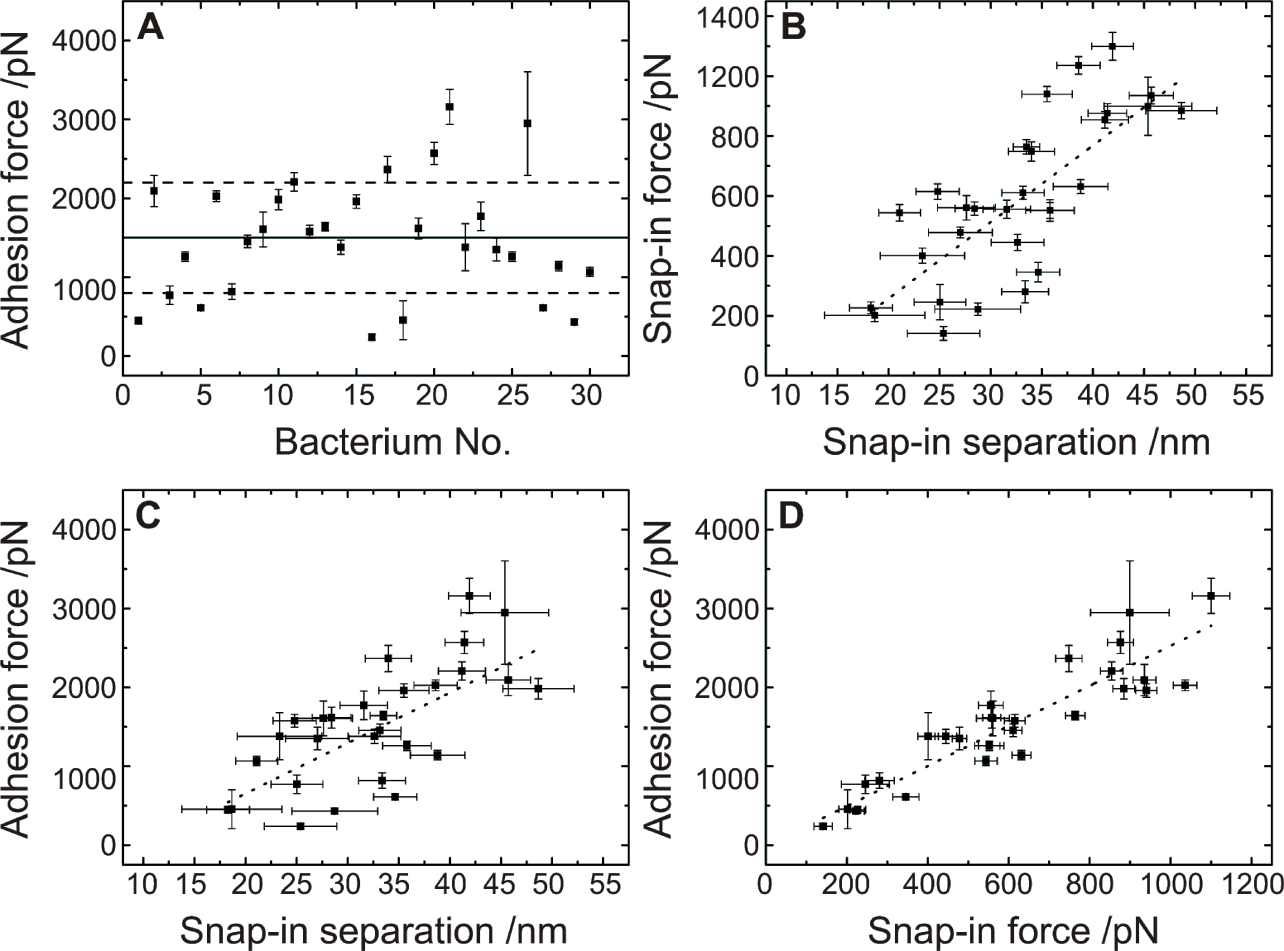
Results of 30 different single cell force spectroscopy experiments on OTS with *S. carnosus* bacterial probes, each data point consists of 30 force/distance curves with a force trigger of 150 pN and a tip velocity of 800 nm/s. A: Solid line: average adhesion force, dashed lines: range of the standard deviation. B–D: Dotted lines: linear fit to the data.

Figure 8A summarizes the results of the measured adhesion forces for 30 different bacterial probes. It contains the OTS-wafer data shown in Figure 7C. For an individual *S. carnosus* bacterial probe on an OTS-wafer, the distribution of adhesion forces is rather narrow, the width of the distribution is typically less than 10% of the average adhesion force, as depicted in the inset of Figure 7A. Comparing different *S. carnosus* probes as shown in Figure 8A, the adhesion forces vary between 400 pN and 3000 pN, with the average at 1500(800) pN (solid line).

Inspecting the approach curve, it is noticeable that also the “snap-in” is an extended event rather than a sudden jump-to-contact (a jump-in or -out can be recorded whenever the gradient of the force exceeds the gradient of the restoring force of the cantilever (i.e., the spring constant) [24]). A closer look at the snap-in events reveals that the snap-in force is proportional to the snap-in separation (Figure 8B) and that the form of the curves greatly resemble each other. Moreover, since the adhesion force is also proportional to both, the snap-in force and -separation, see Figure 8C and Figure 8D, we deduce that the involved mechanisms are identical. Hence also upon approach, cell-wall polymers are involved in establishing the contact to the hydrophobic Si wafer. The snap-in separation reaches values up to 50 nm on hydrophobic surfaces, and can hence serve as an estimate for the hydrodynamic radius of the bacterial cell-wall proteins.

Based on the recently published genome sequence of *S. carnosus* strain TM300 (deposited in the EMBL nucleotide database under accession number AM295250), 19 putative cell-wall anchored proteins harboring LPXTG motifs are predicted, including homologues of well-studied *S. aureus* adhesins such as clumping factor A and B, fibronectin binding protein, and elastin binding protein (cf. [5]). However, nothing is known about the structures and the lengths (e.g., hydrodynamic radius under in vivo conditions) of these putitative cell-wall proteins, making it difficult to correlate the measured adhesion phenomena to specific proteins.

We can now revisit the presented results on the influence of the tip velocity (Figure 5) and the force trigger (Figure 6) to the adhesion process: The long cell-wall polymers need time to come into contact with the surface. The search for contact is a stochastic process; a higher tip velocity thereby results theoretically in a smaller snap-in separation because the residence time in each separation, and therefore the probability that a protein comes into contact in a certain distance, is smaller. Moreover, the polymer needs also time to perform conformational changes in the vicinity of the surface. Both time windows are reduced at higher tip speeds and, hence, the distance at which the cantilever starts to deflect, the snap-in separation, is reduced. The snap-in force is reduced, since the deflection of the cantilever is smaller at the lowest point of the approach curve.

Upon retraction, a variation of the tip velocity probes the rheological properties of the involved group of (stretched) macromolecules, which may also interact collaboratively [41]. Since for the group of cell-wall proteins, no rheological data is available, a prediction for the tip-velocity-dependent behavior is not possible. We find that the adhesion force is constant within the applied variation of tip velocities; moreover, snap-in and snap-off events are highly reproducible. Both together strongly indicate that at these speeds, the macromolecules act elastically. These findings are also in accordance with the study of Alsteens et al. [25], in which “protein nanosprings” are one model description of microbial adhesins.

According to our model of *S. carnosus* adhesion, a higher force trigger should provoke a larger contact area, a closer contact and involve additional cell- wall polymers to tether. All of which should result in a higher adhesion force and a different form of the retraction curve. The snap-in event, however, should not be affected. The experiments reveal that indeed snap-in separation and force are independent of force trigger, c.f. Figure 6B and Figure 6C and the adhesion force is increasing as expected Figure 6A. Also, for each force trigger (as well as for each *S. carnosus* bacterial probe), a characteristic set of force/distance curve can be recorded (Figure 6D).

**Figure 9:**
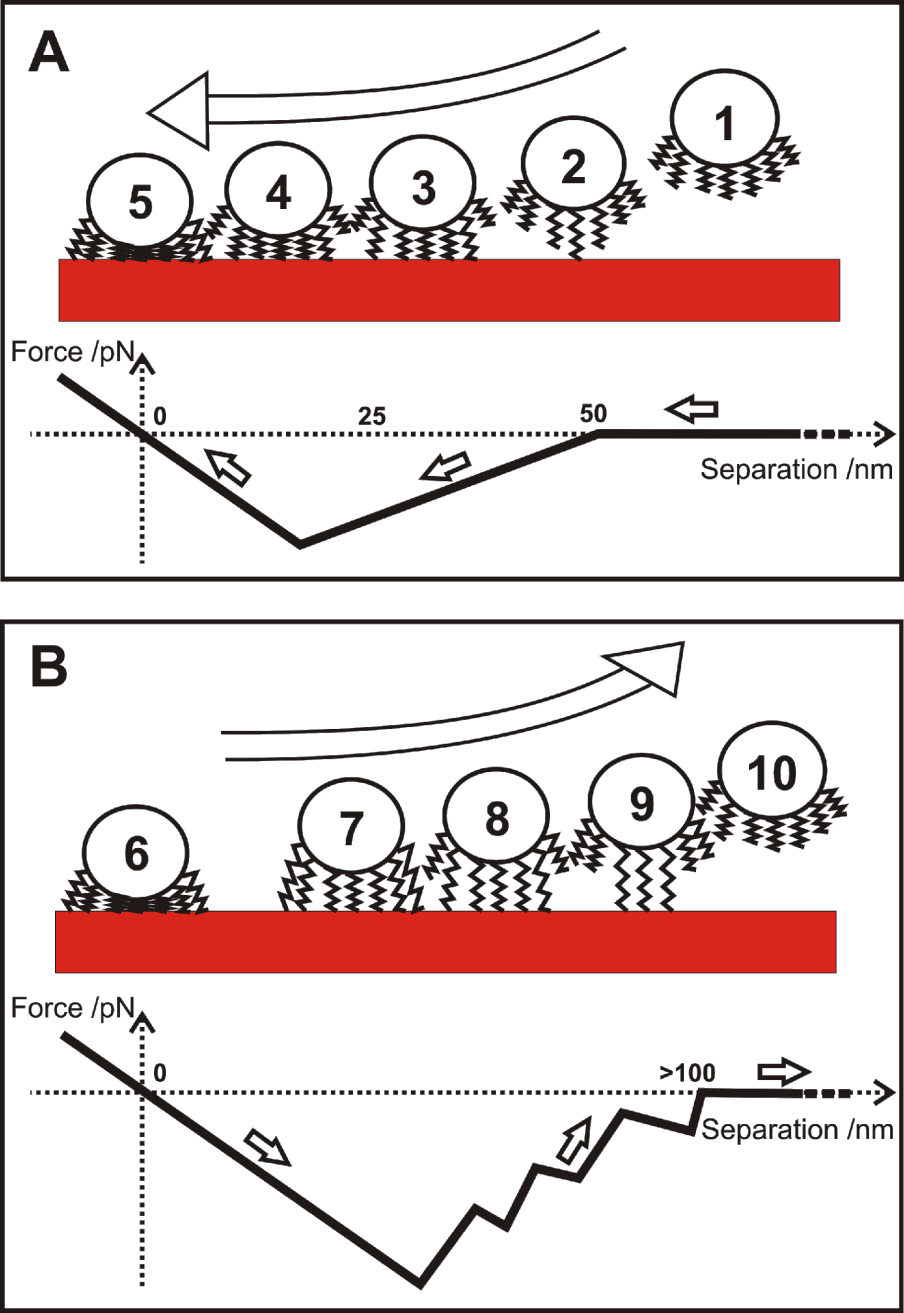
Sketch of approach (A) and retraction (B) of a single bacterial probe and respective force/distance curves. For clarity, neither the AFM cantilever nor the macromolecules that are not involved in adhesion are drawn.

Merging all experimental results, we propose the model sketched in Figure 9: Upon approach, Figure 9A, the cell-wall proteins interact with the surrounding medium (1) and, if at reach, with the surface (2). If an attractive surface is in the vicinity, parts of the proteins can tether (unspecifically in our case) to the surface. Tethering can start at distances below 50 nm, c.f. Figure 8B and Figure 8C. The experiments show that this is the case for hydrophobic wafers. The distance of 50 nm can, hence, serve us as an upper estimate for the coil size of the protein in solution. A further approach gives more proteins the opportunity to tether (3) until a point is reached, at which the maximum attractive force is reached (4). From now on, the proteins start to act as elastic springs that are compressed by the force exerted by the cantilever through the piezo drive. Nevertheless, also in this phase, additional proteins may tether. Approach is stopped shortly after zero force has been reached (5).

Upon retraction, Figure 9B, first, the elastic springs are released (6), achieving the same slope in the curve as during approach. It indicates a reversible fold-and-stretch mechanism of multiple chains. Then, some of the springs start to be stretched against the steric repulsion of the coil (7) [36], followed by a loss of contact of single macromolecules, each of which gives rise to a sudden jump in the force/distance curve (8) and (9) until the entire bacterium has lost contact (10). Depending on type and number of the involved proteins, the retraction curve looks different for every bacterial probe, a fact that has been found earlier in non-bacterial systems involving macromolecules [30,36–38]. For the exact same bacterial probe and the identical contact area (realized by an identical force trigger) and even if 30 approach/retraction cycles have been performed on a different surface, the form of the force/distance curve is characteristic and can be taken as a “fingerprint” for the individual cell.

## Conclusion

To conclude, our experiments strongly corroborate the model that the unspecific adhesion of *S. carnosus* is mainly governed by number, properties and arrangement of the bacterial cell-wall proteins. Through this, the proteins are subject to van der Waals and electrostatic forces as well as forces due to hydrophobic interaction. Comparing hydrophilic and hydrophobic Si wafers (in our case differing only in a monomolecular OTS layer), we find for the exact same bacterial probe strong adhesion of *S. carnosus* to the hydrophobic wafers (up to about 3000 pN) and low adhesion (close to the experimental resolution, about 30–50 pN) to the hydrophilic ones. From that we infer that the hydrophobic interaction is responsible for the strong adhesion on the hydrophobic wafers, exceeding the forces exerted by electrostatic and van der Waals forces by at least an order of magnitude.

The main observations are (i) the form of the force/distance curves is characteristic for each bacterium, (ii) this form is independent of the “adhesive history” and (iii) the retraction curves (including the adhesion forces) are unaffected by the tip velocities probed. These observations lead us to the conclusion that cell-wall proteins act as elastic springs. Since the separation at which the cantilever starts to deflect, the snap-in separation, reaches values up to 50 nm on hydrophobic surfaces, we can estimate the extension of the cell-wall proteins.

Aiming at understanding the detailed form of the force/distance curves, it is inevitable to shed more light onto the “real” molecular composition of the bacterial surface, possibly with the help of atomistic simulations. For future studies, single-cell force spectroscopy can additionally be combined with genetic tools that enable us to specifically modify the composition of the cell-wall proteins. That way, the responsible adhesins can be identified for each of the bacterial species.
